# Rheumatology in Greece: pros and cons

**DOI:** 10.31138/mjr.31.1.8

**Published:** 2020-03-31

**Authors:** Haralampos M. Moutsopoulos

**Affiliations:** Athens Academy, Athens, Greece

**Keywords:** rheumatology, service, training, research, Greece

Rheumatology is a medical discipline which diagnoses and treats patients with autoimmune, inflammatory, infectious, metabolic, genetic, neoplastic, haematologic, endocrine, and gastrointestinal diseases which express symptoms and signs from the musculoskeletal system.^1^

An effectively trained rheumatology specialist should have sufficient education in internal medicine similar to that of rheumatology, an ability to critically evaluate imaging and laboratory tests and, over and above all, an empathetic approach to chronically ill rheumatic-disease patients.^2^

Rheumatology units in Greece have increased exponentially in the last 30 years, particularly in University and private hospitals. At present, outpatient and inpatient rheumatology departments or units function in tertiary care hospitals of the National System of Health (NSH), medical schools and private hospitals, the majority of which train rheumatologists. This had as a result a significant increase in the number of rheumatologists in Greece, which has now reached 3.7 specialists per 100,000 population. In a recent systematic literature review comparing health care system models, it was calculated that the number of rheumatologists needed to serve a population of 100,000 persons, ranges from 0.7 for UK to 3.5 for Spain.^3^ From these data, it appears that our country has a supersufficient number of rheumatologists. Today, 60 young physicians are trainees in rheumatology at NSH and University hospitals. Several of the rheumatology training positions are part of Internal Medicine departments, while the majority is independent out-and in-patient rheumatology departments. The educational programs, as well as the diversity and load of patients in the different departments or units which train rheumatologists, vary. The education of trainees and trained rheumatologists is supplemented through: a) the bi-annual national meetings and the annual “Spring and Winter Rheumatology days”, organised by the Greek Rheumatology Society (ERE); b) the content and educational activities organised by ERE’s official Journal, the Mediterranean Journal of Rheumatology; c) the annual meetings organised by the rheumatology units of the Medical Schools of Ioannina, Patras and Thessaly; and d) the educational program for all rheumatology trainees of our country, which was instituted ten years ago in the Department of Pathophysiology in Athens Medical School. A completed cycle of this program lasts two years, and is held all day every first Saturday of the month. Educators are almost all academic rheumatologists from Greece and abroad, as well as heads of NSH departments or units. This program is popular and highly attended. Trainees take written multiple-choice tests at the end of each session, as well as at the end of the two-year program, to attest the successful completion of the program. Equally, all the educators at the end of the program are evaluated by the trainees.

Is the rheumatology training in our country sufficient and similar to training programs of other European countries? A recent study, using an electronic survey, attempted to answer this question. The authors analysed and compared the educational experience in rheumatology specialty training programs across European countries. They reported that self-ability is generally high; significant differences, however, were noted in the learning structure and assessment of competences. These findings suggested that the educational outcomes may also differ, and concluded that harmonisation of European rheumatology training programs should be undertaken.^4^

I believe that there is room for improvement of the education and training of new rheumatologists in Greece: a) the training period in internal medicine required for the acquisition of the rheumatology specialty is two years with the current training curriculum. Since rheumatic diseases express manifestations from all organs and systems of the body, the time allocated for training in internal medicine should be at least equal to the time allocated to rheumatology; b) the Ministry of Health (MH), in collaboration with ERE, should encourage or even make obligatory the rotation of trainees during their training period from one rheumatology department to another. In this way, the young physicians will have the chance to be exposed to different educators with different level of knowledge, mentality, patient approach, educational ability and infectious enthusiasm to transfer their knowledge,^5^ but also, to a greater variety of patients that will give them the opportunity to be acquainted equally well with handling all sorts of rheumatic disease cases, from regional musculoskeletal to difficult systemic ones; c) in order for the rotation to be beneficial for the trainees the above bodies (MH and ERE), after careful evaluation, should develop educational units consisting of University, NSH and private hospitals, which have rheumatology facilities appropriate for the education of young physicians. It is of paramount importance in our country, to realise the value of periodical evaluation (every 3–5 years) of educational rheumatology units and educators by trainees and vice versa, in order for the quality of provided education to be kept at high standards, as it has been set by the “Union Europeenne des Medecins Specialistes” (UEMS)^6^; and d) last but not least, a national unified educational curriculum which will be followed by any and every center which provides rheumatology training should be developed. Steps towards achieving this have already been taken by relevant sub-committees of ERE.

Despite the limitations and difficulties in the training and educational system for rheumatology in our country, young physicians with sincere desire, drive, perseverance for self-education, devotion to patient care and early exposure to the beauties of research can be superbly educated. Those individuals, upon immigration for further training and experience in another European or transatlantic country, will realise that they are equal or even superior to their contemporary colleagues.

Greece, as described previously, has a significant number of rheumatology sections in the NSH, the Universities and the private hospitals, and a great workforce in rheumatology. The final question which should be addressed is whether rheumatologists in Greece produce new knowledge. To answer this question, a PubMed search was conducted, which compared our productivity with that of other European countries with similar populations, but different Gross Domestic Product (GDP) per capita in US Dollars (USD) (*Table 1*). Greece and Austria, in the same period of time, have produced a similar number of publications, despite the fact that Austria has double the GDP per capita of Greece. The quality of research produced from these countries was not evaluated. Of note, the period during which this rheumatology productivity study was conducted, coincided with the years when Greece experienced a disastrous economic crisis; therefore, the financial resources for research were grossly diminished, and the obstacles to acquire research reagents from abroad were huge. Despite that, should we be proud of our performance? Definitely not! This accomplishment should not let us “rest on our laurels”, but it should encourage and stimulate us for better and more scientific work.

**Table 1.** Number of Publications in the last 10 years in the field of “Rheumatology” from countries with population 9–11×10^5^ and different gross domestic product per capita.

**Country**	**Number of publications**	**Gross Domestic Product per capita (USD)**
Sweden	1477	54000
Austria	699	52000
**Greece**	**546**	**20000**
Portugal	372	23000
Hungary	247	16000
Czechia	195	23000
